# Predator–Prey Interactions Examined Using Lionfish Spine Puncture Performance

**DOI:** 10.1093/iob/obaa049

**Published:** 2021-01-27

**Authors:** K A Galloway, M E Porter

**Affiliations:** Biological Sciences, Nicholls State University, 906 E 1st St, Thibodaux, LA 70301, USA; Biological Sciences, Florida Atlantic University, 777 Glades Road, Boca Raton, FL 33431, USA

## Abstract

Puncture mechanics can be studied in the context of predator–prey interactions and provide bioinspiration for puncture tools and puncture-resistant materials. Lionfish have a passive puncture system where venomous spines (dorsal, anal, and pelvic), the tool, may embed into a predator’s skin, the target material, during an encounter. To examine predator–prey interactions, we quantified the puncture performance of red lionfish, *Pterois volitans*, spines in buccal skin from two potential predators and porcine skin, a biological model for human skin. We punctured dorsal, anal, and pelvic lionfish spines into three regions of buccal skin from the black grouper (*Mycteroperca bonaci*) and the blacktip shark (*Carcharhinus limbatus*), and we examined spine macro-damage (visible without a microscope) post puncture. Lionfish spines were more effective, based on lower forces measured and less damage incurred, at puncturing buccal skin of groupers compared to sharks. Anal and dorsal spines incurred the most macro-damage during successful fish skin puncture trials, while pelvic spines did not incur any macro-damage. Lionfish spines were not damaged during porcine skin testing. Anal spines required the highest forces, while pelvic spines required intermediate forces to puncture fish skin. Dorsal spines required the lowest forces to puncture fish skins, but often incurred macro-damage of bent tips. All spine regions required similar forces to puncture porcine skin. These data suggest that lionfish spines may be more effective at puncturing humans such as divers than potential fish predators. These results emphasize that puncture performance is ultimately determined by both the puncture tool and target material choice. Lionfish puncture performance varies among spine region, when taking into account both the puncture force and damage sustained by the spine.

## Introduction

Puncture performance is important for a variety of reasons including prey acquisition (active systems like teeth, claws, and beaks) and defense (passive systems like spines and thorns) (Spring 1965; Dingle and Caldwell 1978; Collins et al. 1980; Halpern et al. 2007; Whitenack and Motta 2010; DeVries et al. 2012; Galloway et al. 2016). Puncture performance not only depends on the sharpness, shape, and material properties of the tool, but also the target material, the speed of puncture, and the properties of the fluid medium (Anderson 2018; Crofts and Anderson^2018^; Crofts et al. 2019). Likewise, puncture performance can be affected by resistance, resulting from material properties of the target (Zhu et al. 2013; Martini and Barthelat 2016; Boggett et al. 2017). We can examine interspecific relationships by exploring puncture performance among structures (puncture tools) and target materials. Due to the interspecific relationships and close encounters among invasive lionfish and native fish species, lionfish are a model to examine predator–prey puncture mechanics in an ecological framework.

**Fig. 1:**
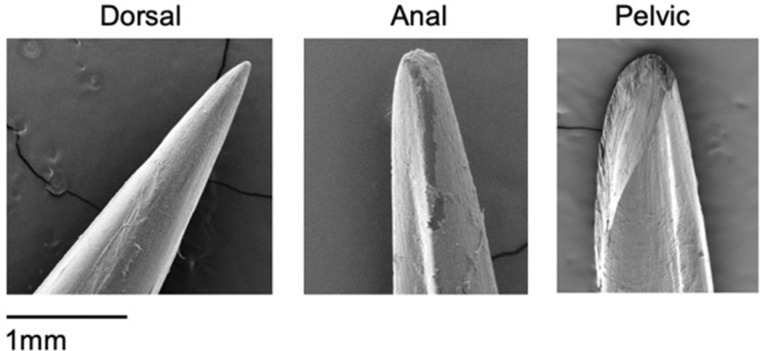
SEM images of dorsal, anal, and pelvic spine tips of *P. volitans*. Scanned at 15 kV and 54× magnification. The anal spine shows unknown darker tissue residue, and the pelvic spine shows micro-damage that still maintains a sharp tip visually. SEM images are from the FAU High School Owls Imaging Lab.

Lionfish have a passive puncture defense system: venomous spines pierce into the skin of moving predators during an encounter (Anderson 2018). Red lionfish (*Pterois volitans*) have 18 tri-lobed venomous spines spanning three fin locations: 13 dorsal, 3 anal, and 1 on each pelvic fin (Halstead et al. 1955; Galloway and Porter 2019). The dorsal spines are long relative to their body size. The anal and pelvic spines are shorter and slightly recurved compared to the dorsal spines, but they can absorb more elastic energy and are stiffer structures (Galloway and Porter 2019). The spines have grooves along the majority of their lengths serving as the pathways for venom delivery. The distal tips of spines, used in initial puncture, lack grooves (Fig. 1). Lionfish spines are capable of inflicting damage in the oral cavities of predators, and injuring divers and fishermen (Patel and Wells 1993; Muñoz 2017).

Lionfish (*P. volitans*) are now an established invasive species in the Western Atlantic (Côté et al. 2014). Although lionfish have no significant predators in their invasive range, larger predators such as groupers and sharks may eventually recognize lionfish as a consistent food source. When sharks or grouper species predate on lionfish, they often immediately retreat without any notable injury to the lionfish (Albins and Hixon 2013). Although it is unknown whether predator oral cavities are damaged by lionfish consumption. Stingray spines are composed of mineralized collagen, similar to lionfish spines, and they have been shown to cause mortality in some mammals such as killer whales and fur seals, but does not affect other species such as wedgefish (Duignan et al. 2000; Dean et al. 2017; Hocking et al. 2020). Humans are considered lionfish predators and are currently the only significant biological control for eradicating this invasive species (Côté et al. 2014). This study is the first to examine mechanical interactions between lionfish defensive structures (puncture tool) and predator skin (target material).

The goal of this study was to determine the puncture performance, and spine macro-damage of the venomous spines of the red lionfish, *P. volitans*, in target materials from potential predators (black grouper and blacktip shark) and human skin, using porcine skin as a model, because of the incidence of human injury due to lionfish encounters during water sports (Côté et al. 2014). We chose these fish skin regions (braincase, hyoid, and upper jaw) to examine puncture performance (puncture force [N] and input energy [Nmm]) outside and inside the buccal cavity of both bony and cartilaginous species representing potential lionfish predators. We hypothesized that lionfish spines will puncture grouper buccal skin with less force and incur less damage compared to shark buccal skin, due to the presence of dermal denticles on shark skin. Specifically, we hypothesized that lionfish spines will be less effective when puncturing the shark upper jaw region due to the known density of dermal denticles in this region. Lionfish have been shown to damage human skin (specifically the long dorsal spines). We hypothesized that lionfish will most effectively puncture (lowest forces and least damage) porcine skin because it has additional adipose and muscle tissue layers, which have been shown to be less resistant to puncture (Atkins 2009). We hypothesize that dorsal spines will require higher forces to puncture all skin regions (porcine and fish) and incur the most macro-damage because they are less stiff and absorb less energy than the anal and pelvic spines (Galloway and Porter 2019). Finally, we expect that sharks will be the most effective predator because dermal denticles on their skin will result in more lionfish spine damage, and require higher puncture forces compared to groupers and humans.

## Methods

### Spine and skin preparation

We obtained dead adult lionfish (*P. volitans*) specimens from local derbies in South Florida, USA. Lionfish are invasive and no permit is required for fishing this species in the state of Florida (Florida Fish and Wildlife Conservation Commission). We then selected, removed, and measured spines based on our previous methods (Galloway and Porter 2019). Using a dissecting microscope, any spines with damage were excluded from this study. For each adult lionfish specimen (*n* = 34 fish; *N* = 102 spines; total length = 238–316 mm), the fourth dorsal, left pelvic, and third anal spines had respective spine lengths ranging from 55 to 84 mm, 20 to 32 mm, and 13 to 27 mm.

**Fig. 2:**
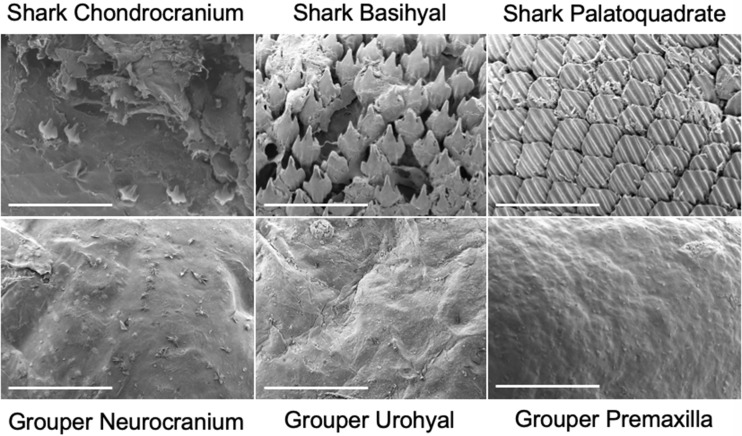
SEM images of blacktip shark and grouper buccal skin regions: braincase (chondrocranium; neurocranium), hyoid (basihyal; urohyal), and upper jaw (palatoquadrate; premaxilla). Scanned at 10 kV and 40× magnification. Scale bar, 500 microns. SEM images are from the FAU High School Owls Imaging Lab.

**Fig. 3:**
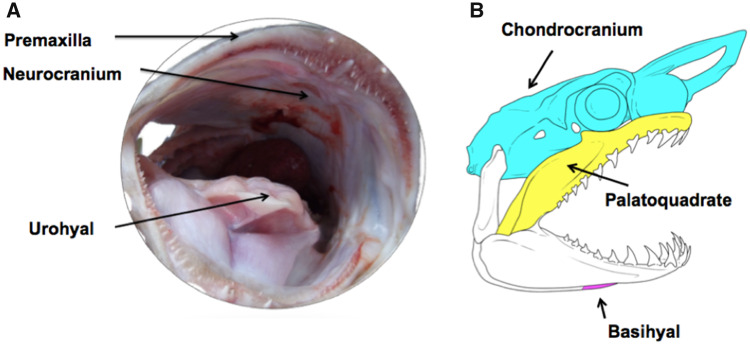
(**A**) Black grouper, *M. bonaci* and (**B**) blacktip shark, *C. limbatus*, buccal regions: braincase (chondrocranium; neurocranium), hyoid (basihyal; urohyal), and upper jaw (palatoquadrate; premaxilla). The upper jaw regions (palatoquadrate and premaxilla) are the only outer skin regions. All other skin regions are dissected from inside the buccal cavity. Shark jaw image adapted from Moss (1984); Illustration credit: Ivana Heerdegen.

Large grouper and shark species are appropriate target materials to examine for this study because they have been documented to occasionally consume lionfish (Albins and Hixon 2013). Skin micro-morphology differs greatly between bony and cartilaginous fish, which have dermal denticles (Fig. 2). We obtained a sufficient sample size of blacktip sharks, *Carcharhinus limbatus* (*n* = 9 fish; fork length = 132–145 cm; all males) through National Oceanic and Atmospheric Administration (NOAA) fishing trips spanning from Georgia to North Carolina. We collected adult black grouper heads (*Mycteroperca bonaci*; *n* = 9 fish; head length = 262–285 mm; sex of fish could not be determined) from local fish markets. For each grouper and shark, we dissected skin from three different oral locations (Mumby et al. 2011; Fig. 3). Analogous skin regions of the oral cavity were used in each species: upper jaw (premaxilla in grouper and palatoquadrate in shark), bottom of braincase (neurocranium in grouper and chondrocranium in shark), and hyoid region (urohyal in grouper and basihyal in shark) (Fig. 3). Within each skin region, we dissected two adjacent samples from each shark and grouper to obtain enough buccal skin for our experimental design (*n* = 15 mechanical tests per skin region). All grouper buccal skin samples had ∼ 2–3.5 mm of underlying connective tissue present. All shark buccal skin samples had ∼2–4 mm of underlying connective tissue present.

Mechanical property data from fish skin have largely focused on the external body, rather than the buccal regions, but dermal denticle density has been shown to affect the stiffness and toughness of the external skin (Naleway et al. 2016; Wainwright and Lauder 2016 ; Szewciw and Barthelat 2017; Ankhelyi et al. 2018; Creager and Porter 2018; Kenaley et al. 2018). We dissected 12 mm samples from five sharks for each buccal region (*n* = 15 images) to calculate average dermal denticle density (1 mm^2^) using scanning electron microscopy (SEM; JCM-6000Plus; 10 kV and 40× magnification; Fig. 2). We also used SEM to image the same buccal regions for grouper skin.

We obtained adult porcine abdominal skin (Sierra Medical; Whittier, CA, USA) and froze the skin immediately upon arrival. Porcine samples included a 2 mm layer of skin, a 1–2 mm layer of underlying fat, and a 1 mm layer of muscle. We kept all skin (fish and porcine) frozen and we thawed samples once to dissect out squares (2.54 × 2.54 cm) and then immediately used samples in puncture testing. To determine testing order, spines (*n* = 3) from individual lionfish (*n* = 49) and skin samples from shark, grouper, and pig we used the random number generator in Excel.

### Puncture testing

We placed lionfish spines in tension clamps on an Instron E1000 (Norwood, MA, USA), all electric dynamic test instrument, at a 90º angle, using a 250 N load cell. All spines (dorsal, pelvic, and anal) were clamped at 50% of their total length. We placed skin samples on top of sandpaper to prevent slippage and on top of a rubber block, which provided more traction than the metal Instron platen (Galloway et al. 2016). The actuator moved the spines into the skin at a rate of 10 mm min^−1^. These settings are within a wide range of speeds used in puncture testing of fish scales and skins, and we needed a consistent speed across all skin samples in order to compare puncture forces (Yang et al. 2014; Galloway et al. 2016). A biologically realistic speed of a grouper or shark strike may not allow for the machine to capture initial puncture, or damage the spine prior to puncture. Shark tooth velocity has been estimated as fast as 0.15–5.5 m s^−1^ and goliath groupers are known to be capable of short explosive bursts of speed (Bullock and Smith 1991; Corn et al. 2016).

Tests terminated at a 10% load drop, capturing the initial puncture or load drop, which is the force (N) required to initially puncture the material or the moment the spine tip broke through the flesh (Bergman et al. 2017; Galloway et al. 2016). Puncture testing at slow speeds allows for the test to be terminated at a specific load drop. At faster speeds, tests need to be terminated at the puncture tool length/puncture material height and validated via high speed video recordings (Bergman et al. 2017). Buckling was not observed in any spines including the longer dorsal spines, which were a concern due to the longer spine length. Buckling would be seen on the force/displacement curve if the slope of the line started to flatten once a certain load threshold is achieved, instead of the distinct 10% load drop (or a sharp decrease of the slope) indicating initial puncture. In biological materials, buckling has been described in lordotic vertebrae of sea bass (Kranenbarg et al. 2005).

**Fig. 4:**
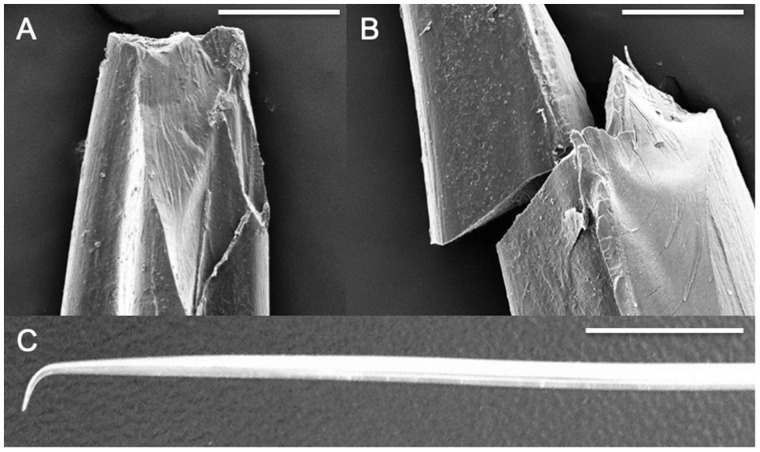
Micro and macro-damage of *P. volitans* spines post puncture testing. Scanned at 15 kV and 65× magnification. (**A**) Micro-damage at tip of anal spine. Scale bar, 200 microns. (B) Micro-damage of dorsal spine still intact 5 mm below the tip. Scale bar, 200 microns. (C) Macro-damage of dorsal spine, resulting in bending at the tip. Scale bar, 1 cm. SEM images are from the FAU High School Owls Imaging Lab.

We measured the extension or puncture depth (mm), to calculate input energy (Nmm), which is defined simply as the product of force and the distance in the direction of the force (Anderson et al. 2019). The extension or puncture depth was measured when the test terminated at a 10% load drop. When the spine was still embedded into the skin, we marked the spine with a Sharpie pen to verify the puncture depth measurement from the Instron. We then returned the spines to the original testing position, withdrawing the spines from the skin, and all successful punctures of the skin were verified under a dissection microscope. We defined micro-damage as damage that was not visible to the naked eye, but was apparent under light microscopy or SEM (Figs. 1 [pelvic], 4A and B). We defined lionfish spine macro-damage as visible damage (bending or fracturing such as visible spine fragments in the skin) that occurred after puncture testing (Fig. 4C). In this study, we quantified spine damage on a macro-scale because it would likely have effects on the puncture tool during future feeding events.

### Statistics

All statistical tests were done using JMP (SAS Institute Inc., NC, USA), and all tests had sufficient sample sizes to conduct the analyses and were significant when *P* < 0.05. For all ANOVA models, data were analyzed for normality and homoscedasticity, and no transformations were necessary.

We used a one-way ANOVA to determine if there was a significant difference in dermal denticle density among shark skin regions (upper jaw, braincase, and hyoid). To evaluate lionfish puncture forces, we used a two-way ANOVA with puncture tool (spine region: dorsal, pelvic, and anal) and puncture material (grouper upper jaw, grouper braincase, grouper hyoid, shark upper jaw, shark braincase, shark hyoid, and porcine abdomen) as main effects, and we examined the interaction between spine region and skin region. We omitted puncture data from the shark upper jaw region before statistical analyses, because only two lionfish spines (one anal and one pelvic) out of 15 tested, effectively punctured this skin type. This same statistical method was used to evaluate input energy. For the grouper and the shark, we used 15 spines per skin region: 5 dorsal, 5 pelvic, and 5 anal from 25 lionfish, while for the porcine abdomen skin we used 27 spines: 9 dorsal, 9 pelvic, and 9 anal from 9 lionfish.

## Results

### Spine and skin damage

**Fig. 5:**
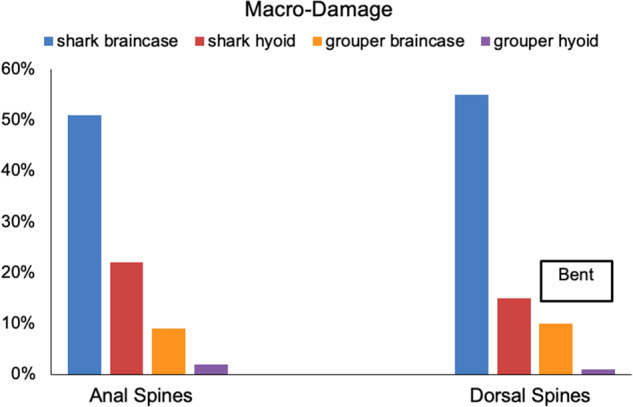
Macro-damage of lionfish spines from fish buccal regions. Anal and dorsal spines incurred the most macro-damage from puncturing shark braincase skin. Anal and dorsal spines incurred the least macro-damage from grouper hyoid skin. These data do not include the unsuccessful puncture data (13 spines) of the shark upper jaw region. The six bars that are not labeled bent, indicate fractured spine tips. All lionfish (*P. volitans*) pelvic spines were able to puncture grouper and shark skin without any visible macro-damage and are not displayed on this figure. Porcine and grouper upper jaw skin are not included in this graph because lionfish spines did not incur any macro-damage of these skin regions during puncture testing.

All lionfish spines (100%) were able to puncture grouper skin. Lionfish spines did experience macro-damage from puncture testing of the braincase and hyoid regions of grouper. Puncture tests for grouper upper jaw skin did not cause any damage to spines (Fig. 5). In grouper skin, ∼11% of spines (all dorsal) bent at the tip and 11% of spines (all anal) fractured at the tip (Fig. 5). Lionfish spines were only able to puncture shark skin in 71% of tests. Only 2 (1 anal and 1 pelvic) out of 15 lionfish spines, or 13%, could effectively puncture the shark upper jaw skin (8.5 N and 10.4 N of force). Since this region was largely resistant to lionfish spine puncture, we do not include it in the remaining analyses. In shark skin, 70% of dorsal and 73% of anal spines fractured at the tip. Lionfish spine tips incurred more macro-damage from shark skin puncture tests compared to grouper skin, supporting our hypothesis. Specifically, more macro-damage occurred in the shark braincase region (Fig. 5). Pelvic spines did not incur any macro-damage in shark or grouper buccal skin (Fig. 5). Lionfish spines did not incur any macro-damage during porcine skin testing.

### Dermal denticle density

SEM was used to quantify dermal denticle density from the three skin regions (*n* = 5 per region) in the shark (Fig. 2). A one-way ANOVA examining denticle dermal density was significant among skin regions (*F*_2,14_ = 827.6, *P* < 0.0001), and *post hoc* Tukey’s tests showed that denticle dermal density significantly differed among all skin regions (*P* < 0.0001). We found that the braincase region had an average of 5.6 dermal denticles/mm^2^, the hyoid region had 41.4 dermal denticles/mm^2^, and the upper jaw had 45 dermal denticles/mm^2^.

Surprisingly, the region with the least amount of dermal denticles did not correspond to less spine damage from puncture testing. Despite having significantly fewer dermal denticles, shark skin from the braincase caused more damage to spines compared to hyoid skin (Fig. 5). Upper jaw skin was the only region without spacing between denticles, and 87% spines could not puncture this region, supporting our hypothesis. A similar analysis could not be done on grouper buccal skin due to the absence of scales in this area (Fig. 2).

### Puncture force and input energy

**Fig. 6:**
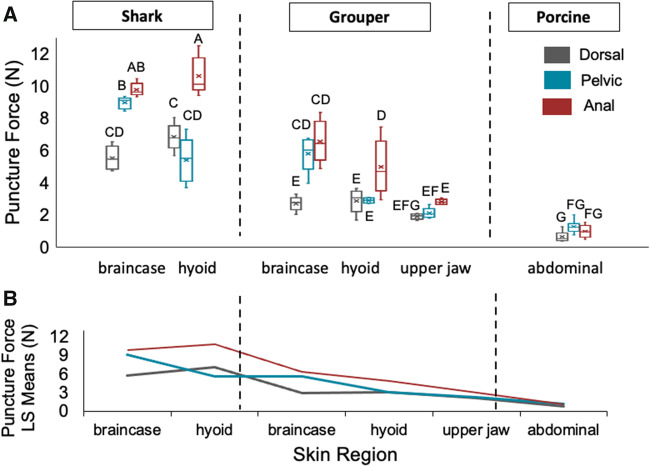
(**a**) Puncture force (N) of *P. volitans* spines (*N* = 102 spines) varied significantly (*F*_17,101_ = 108.61; *P* < 0.0001) among spine regions (*F*_2,101_ = 92.54; *P* < 0.0001) and skin regions (*F*_5,101_ = 304.07; *P* < 0.0001). The interaction term between spine region and buccal region was significant (*F*_10,101_ = 17.05; *P* < 0.0001). On average, anal spines required the highest forces to puncture, pelvic spines intermediate forces, and dorsal spines required the lowest forces to puncture. Shark buccal skin regions (basihyal, chondrocranium) required higher puncture forces on average compared to grouper buccal regions. Box plots represent median, quartiles, and range. Each shark and grouper box represents five spines, and each porcine box represents nine spines. The x denotes the mean. Columns sharing the same letter are statistically similar. Full Tukey’s *post hoc* reports of main effects and interaction terms are in Tables 1 and 2. (**B**) Interaction plot between spine and skin regions. The interaction is between all spines for the porcine skin region, and between the dorsal and pelvic spines (the lines that cross each other) for hyoid and upper jaw skin regions, not the anal spines or the cranium skin regions.

A two-way ANOVA examining puncture force (N) was significant (*F*_17,101_ = 108.61; *P* < 0.0001) among spine regions (*F*_2,101_ = 92.54; *P* < 0.0001) and skin regions (*F*_5,101_ = 304.07; *P* < 0.0001). The interaction term between spine region and buccal region was significant (*F*_10,101_ = 17.05; *P* < 0.0001). Tukey’s *post hoc* tests of main effects showed that anal spines required the highest puncture forces, pelvic spines intermediate forces, and dorsal spines had the lowest puncture forces in skin from all three animals (Table 1). These data refute our hypothesis that dorsal spines would require higher puncture forces. Tukey’s *post hoc* tests of main effects also showed that the porcine skin required significantly lower puncture forces compared to the other skin regions, supporting our hypothesis that this region would be easiest to puncture due to the adipose and muscle layers (Table 1). Puncture forces were similar in the braincase and hyoid regions of the shark, and higher than all other skin regions, supporting our hypothesis that shark skin would require higher puncture forces (Table 1). Tukey’s *post hoc* tests of the interaction term showed that anal spines required more force to puncture buccal skin compared to dorsal spines in all regions, except for the grouper upper jaw skin (Fig. 6A and  Table 2). The upper jaw of the grouper required similar forces to puncture compared to porcine skin (Fig. 6A and Table 2).

**Table 1:** Tukey’s *post hoc* ordered letters report for two-way ANOVA main effects

Main effect	*Post hoc* comparisons puncture force	*Post hoc* comparisons input energy
Spine region		
Anal	A	A
Pelvic	B	B
Dorsal	C	B
Skin region		
Shark braincase	A	A
Shark hyoid	A	A
Grouper braincase	B	B
Grouper hyoid	C	B
Grouper upper jaw	D	C
Porcine	E	D

For each main effect, columns sharing the same letter are statistically similar.

**Table 2:** Tukey’s *post hoc* ordered letters report for significant spine region by skin region interaction term for puncture force (N) and input energy (Nmm) shown in Figs. 6 and 7

Spine region * skin region interaction	*Post hoc* comparisons puncture force	*Post hoc* comparisons input energy
Anal*shark hyoid	A	A
Anal*shark braincase	AB	AB
Pelvic*shark braincase	B	B
Dorsal*shark hyoid	C	C
Dorsal*shark braincase	CD	C
Pelvic*shark hyoid	CD	C
Anal*grouper hyoid	D	C
Anal*grouper braincase	CD	C
Pelvic*grouper braincase	CD	CD
Anal*grouper upper jaw	E	DE
Pelvic*grouper hyoid	E	EF
Dorsal*grouper hyoid	E	EF
Dorsal*grouper braincase	E	EF
Dorsal*grouper upper jaw	EFG	EF
Pelvic*grouper upper jaw	EF	EF
Pelvic*porcine	FG	EF
Anal*porcine	FG	EF
Dorsal*porcine	G	F

Columns sharing the same letter are statistically similar.

An interaction plot displays interactions between corresponding spine and skin regions. Overlapping lines occur between the dorsal and pelvic spines in all skin regions except the shark and grouper braincase (Fig. 6B). Anal, dorsal, and pelvic spines all overlap on the interaction plot for porcine abdominal skin (Fig. 6B). Therefore, there is no interaction among spines and skin for cranial regions in shark and grouper, and for anal spines in fish skin (Fig. 6B).

**Fig. 7:**
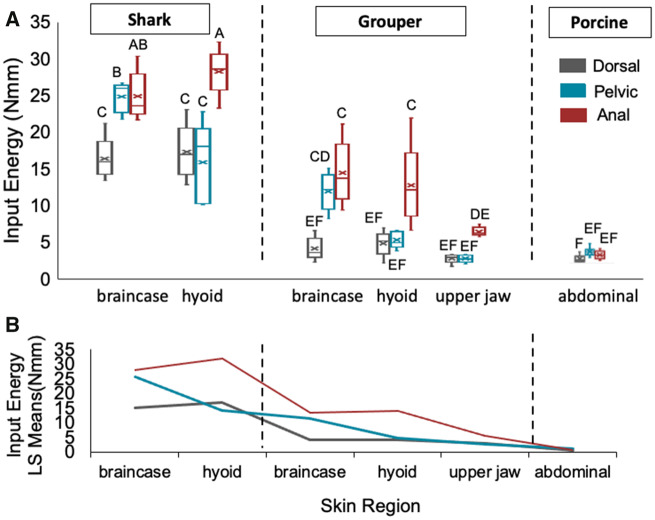
(**a**) Input energy (Nmm) of *P. volitans* spines (*N* = 102 spines) varied significantly (*F*_17,101_ = 83.39; *P* < 0.0001) among spine regions (F_2,101_ = 72.4; *P* < 91.1) and skin regions (*F*_5,101_ = 229.98; *P* < 0.0001). The interaction term between spine region and buccal region was significant (*F*_10,101_ = 15.26; *P* < 0.0001). On average, anal spines required more input energy compared to dorsal and pelvic spines. Shark buccal skin regions (chondrocranium, basihyal) required the highest input energy. Box plots represent median, quartiles, and range. Each shark and grouper box represents five spines, and each porcine box represents nine spines. The x denotes the mean. Columns sharing the same letter are statistically similar. Full Tukey’s *post hoc* reports of main effects and interaction terms are in Tables 1 and 2. (**B**) Interaction plot between spine and skin regions. The interaction is between all spines for the porcine skin region, and between the dorsal and pelvic spines (the lines that cross each other) for hyoid and upper jaw skin regions, not the anal spines or the cranium skin regions.

A two-way ANOVA examining input energy (Nmm) was significant (*F*_17,101_ = 83.39; *P* < 0.0001) among spine (*F*_2,101_ = 72.4; *P* < 91.1) and skin regions (*F*_5,101_ = 229.98; *P* < 0.0001). The interaction term between spine and buccal region was significant (*F*_10,101_ = 15.26; *P* < 0.0001). Tukey’s *post hoc* tests of main effects showed that anal spines required higher input energy, pelvic intermediate, and dorsal spines lowest input energy (Table 1). Input energy was similar in the braincase and hyoid regions of the shark, and higher than all other skin regions (Table 1). The porcine region required the lowest input energy (Table 1). Tukey’s *post hoc* tests of the interaction term showed that anal spines required more input energy than dorsal and pelvic spines for grouper and shark hyoid regions (Fig. 7A and  Table 2).

Similar to puncture forces, an interaction plot shows overlapping lines between the dorsal and pelvic spines in all skin regions except the shark and grouper braincase regions (Fig. 7B). All spine regions overlap on the interaction plot for porcine abdominal skin (Fig. 7B). Therefore, there is no interaction among spines and skin for cranial regions in shark and grouper, and for anal spines in fish skin (Fig. 7B).

## Discussion

Here, we determined the puncture performance of the venomous spines of invasive lionfish (*P. volitans)* in target tissues from potential predators in their established range in the Western Atlantic and Caribbean. Supporting our comparative skin hypothesis, we found that lionfish spines were more effective (lower forces, lower input energy, and less damage) at puncturing grouper skin compared to shark skin (Fig. 5 and Table 1). In shark skin, spines on average punctured at a higher range of forces and sustained large amounts of macro-damage (Figs. 5 and 6A; Table 1). Only two spines, of 15 tests, were able to puncture the shark upper jaw region, supporting our shark skin hypothesis that this region would resist puncture due to the density of dermal denticles in this area. In contrast, lionfish spines easily pierced porcine skin without incurring any macro-damage, supporting our porcine skin hypothesis. Lionfish pelvic spines did not incur any macro-damage for all buccal skin regions (Fig. 5). Anal spines required the highest forces on average to puncture shark hyoid skin, but similar forces as dorsal and pelvic spines to puncture porcine skin (Fig. 6A), emphasizing that target material is a contributing factor to puncture performance. Dorsal spines required the lowest forces to puncture porcine and fish skin, refuting our spine hypotheses (Fig. 6A and Table 1). From our results, we hypothesize a form–function relationship in nature: lionfish anal spine tips fracturing and/or dorsal spines bending and breaking in buccal skin could be biologically advantageous for lionfish to prevent themselves from becoming entangled in predator oral cavities due to lionfish having multiple lines of defense—spines on three fin locations.

### Puncture tool

During our puncture trials, macro-damage still created a visually sharp point at the lionfish spine tip 65% of the time, and this may not affect the lionfish’s ability to defend itself in future encounters. Although we did not quantify micro-damage in this study, we did document it in a pelvic spine when using SEM (Fig. 1). In nature, puncture tools such as teeth usually require some mechanism to counteract dulling, unless they are continually replaced. Replacement teeth of sandbar and tiger sharks require less force to puncture than functional teeth, supporting the theory that shark teeth are regularly replaced to maintain sharpness (Bergman et al. 2017). Rodent teeth are designed so that softer dentine is worn away faster than the hard enamel, which allows for new sections of the enamel material to continuously be exposed (Meyers et al. 2008). It also has recently been shown that sea urchin teeth have a self-sharpening mechanism, which is accomplished through plate chipping (Espinosa et al. 2019). Future studies on lionfish spines could examine possible self-sharpening mechanisms and quantify the effects or patterns of spine dullness.

Spine damage may accumulate or worsen in future feeding events, and anal spines fracturing may be more advantageous in defense, rather than bending as in dorsal spines. Although the longer dorsal spines required less force to puncture fish buccal skins (Fig. 6A), these spines often incurred macro-damage of bent tips (Figs. 4C and 5). In cases where dorsal spines bent after initial puncture, the buccal skin target material did not incur any significant damage, and we suggest that this bent tip would eventually break off or fracture in nature. Bent tips of the dorsal spines only occurred in grouper buccal puncture trials. This may be due to the mucous coating that was visibly present in the groupers obtained, potentially providing less friction during puncture. Dermal denticles of shark skin may provide more friction, preventing bending. Bent tips of dorsal spines may be directly related to the mechanical properties; we have shown that dorsal spine tips have a significantly lower stiffness and absorb less elastic energy than the anal and pelvic spine tips (Galloway and Porter 2019). We suggest that although anal spines required overall higher puncture forces compared to dorsal spines, the type of macro-damaged sustained by anal spines (fracturing) may be more advantageous in future defense compared to the type of dorsal spine tip damage (bending). If the spine is fractured and not bent, the spine may still be able to have a sharp point and puncture, delivering venom. Overall, our data suggest that if grouper and reef sharks recognized invasive lionfish as a consistent food source, predation events over time (especially from sharks), may cause significant damage to the tips of lionfish spines (particularly the highly bendable dorsal spines), and affect future defense capabilities of those individuals.

There may be a mechanical trade-off between spine strength and sharpness for lionfish. We hypothesize that all lionfish spines are considered to be relatively sharp puncture tools because they all were able to puncture porcine skin. Similarly, only cactus spines that were classified as being sharp were able to puncture porcine tissue (Crofts and Anderson^2018^). In our data, lionfish spines often fractured at the tip post puncture which embedded spine fragments into rougher materials such as shark buccal skin. Based on these observations, we hypothesize that the mineralized collagenous material of the spine is not strong. Lionfish spine tips fracturing in tough materials could be biologically advantageous for lionfish to prevent themselves from becoming entangled in predators with tough skin. It may be more beneficial for lionfish spines to easily bend and fracture during an encounter with a predator, because lionfish (*P. volitans*) have many defensive tools: 18 spines spanning 3 fin locations.

Spine curvature, the presence of grooves, and mineralization may all affect mechanical properties and puncture performance. Both pelvic and anal spines are stiffer and can store more elastic energy compared to dorsal spines (Galloway and Porter 2019). Pelvic and anal spines do not bend as much as dorsal spines perhaps making them more effective at puncture. We hypothesize that differences among pelvic and anal spine curvature along the length, may affect the puncture performance in different target materials. Another possibility is that the pelvic spine grooves extend further than the anal spine grooves causing differences in morphology, and perhaps sharpness (Halstead et al. 1955). The presence of grooves extending further in pelvic spines may be a contributing factor explaining their lack of macro-damage in this study. Future studies could quantify metrics of sharpness and the amount of mineralization along the spine length and among regions. Differences in spine mineralization can lead to differences in mechanical properties and sharpness, which can then directly affect puncture performance.

### Target material

Previous research has suggested that shark tooth morphology is not necessarily a good predictor of biological role, suggesting an indirect relationship between form and function in this puncture system (Whitenack and Motta 2010). Similarly, our study shows an indirect relationship between form and function of puncture performance and armored target materials. Shark dermal denticle density is not a direct predictor of lionfish puncture performance and spine damage. Hyoid regions of shark skin had a significantly higher dermal denticle density (41.4 dermal denticles/mm^2^) compared to the braincase regions (5.6 dermal denticles/mm^2^), but resulted in similar puncture forces and input energy among spine regions (Table 1). Skin from the braincase caused more damage to spines compared to hyoid skin, despite having fewer dermal denticles (Fig. 5). We hypothesize that braincase skin may be tougher because it is protecting nervous and olfactory tissues, compared to the hyoid skin which is protecting the hyoid, lower jaw, and muscles.

Shark dermal denticles are continually replaced, which also could affect the puncture ability, and may explain the two lionfish spines that were able puncture the upper jaw region. Our data suggest that the material properties of the shark buccal skin underneath the denticles are also an important factor to consider, when there is significant spacing between denticles. For example, the high tensile strength of stratum compactum in striped bass contributes to the puncture resistance (Szewciw and Barthelat 2017). Detailed histology of the stratum layers inside and outside the oral cavity for both large bony fish and cartilaginous predators could contribute to our knowledge of skin puncture resistance.

Puncture performance is affected by the target material the structure is piercing. Anal spines required the most force to puncture shark hyoid buccal skin, but similar forces as pelvic and dorsal spines to puncture porcine skin (Fig. 6A and Table 2). These data highlight that puncture performance is dependent on the target material, and not just the puncture tool material properties. This was important to take into account in this study, due to the great differences in micro-morphology of the shark, grouper, and porcine skins (Fig. 2; Supplementary Fig. S1). When examining puncture-resistant materials, surface variations and spacing should be accounted for because dermal denticle density did statistically differ among all shark skin regions. We emphasize that for each study examining puncture performance, the choice of target material is equally important as the material composition of the puncture tool, and it should be biologically relevant to the specific study. This is also applicable when examining potential biomimetic applications in materials that do not mimic the natural interactions.

### Interspecific interactions

Predator–prey puncture mechanics is more than just the influence of the puncture tool; it is a complex interaction between the tool and the target material that the predator encounters (Grisley et al. 1996; Whitenack and Motta 2010; Galloway et al. 2016). This is highlighted in the interaction plots, showing interactions only between the puncture tool and target material for the dorsal and pelvic spines in the hyoid and upper jaw regions, and for spines in the porcine skin (Figs. 6B and 7B). Few studies have focused on passive puncture systems, where the target material applies force to the tool often during a defensive movement. Our data suggest that groupers may be affected by puncture wounds from lionfish spines, whereas reef shark species may not be affected substantially with respect to the predator’s oral anatomy, due to higher puncture forces needed and observations of spine macro-damage. Previous research has shown that the wedgefish (*Rhynchobatus*) often feed on stingray species despite oral damage from their spines (Dean et al. 2017). Therefore, lionfish spines may not be a major deterrent of predation after all.

It is also important to remember that the lionfish venom may affect the puncture wounds and could worsen the mechanical damage. Future studies could determine the amount of the grooved portion of the spine that is required to embed into a predator for a sufficient amount of venom to be released, and how long it takes for the venom to be replaced. Prey defense systems such as venomous spines often co-evolve with the morphology of predators. Studies such as these are important because it has been hypothesized that predators (sharks, groupers, and eels) could eventually recognize invasive lionfish as prey, and act as a biological control (Albins and Hixon 2008; Mumby et al. 2011; Muñoz 2017). Currently, the only significant control for lionfish is humans spearfishing.

### Puncture resistance and other applications

Here, we determined the puncture performance of lionfish spines in biologically relevant target materials, and we demonstrated that the upper jaw region in blacktip sharks can effectively resist puncture. This region also had the highest dermal denticle density on average and did not have any spaces or gaps between denticles (Fig. 2). Skin can be puncture resistant due to armor, such as shark denticles, or loose skin attachments to muscles, as in hagfish (Zhu et al. 2013; Martini and Barthelat 2016; Boggett et al. 2017). Determining puncture resistance of marine vertebrate skin such as sharks, hagfish, and striped bass has led to innovations in biomimetic design and numerous patents (Sullivan 1982; Sundnes 2011; Zhu et al. 2013; Martini and Barthelat 2016; Boggett et al. 2017).

Future studies could benefit from using lionfish spines in designing materials for medical purposes, because they appear to be incapable of puncturing armored materials, but effective at puncturing unarmored material such as porcine and human skin. This is surprising because the lionfish defense mechanism has probably co-evolved with their armored predators. Lionfish spines may be useful in creating reusable syringe needles or plungers that can be sterilized and refilled, which would decrease biomedical waste and disposal costs (Galloway and Porter 2019). Due to the lionfish spine cross-section and the venom delivery being located along the length of the spines, we suggest future studies on modifying the mineralized collagenous material to strengthen the structure and investigate biomimetic applications. Future studies could investigate if the spine grooves or modification to the grooves would be sufficiently effective at injection in a medical setting.

## Supplementary Material

obaa049_Supplementary_Data
