# Supplementary figures and images for: Predator–Prey Interactions Examined Using Lionfish Spine Puncture Performance
Source: Integr Org Biol. 2021 Jan 27;3(1):obaa049. doi: 10.1093/iob/obaa049 (PMC7840115; doi:10.1093/iob/obaa049)

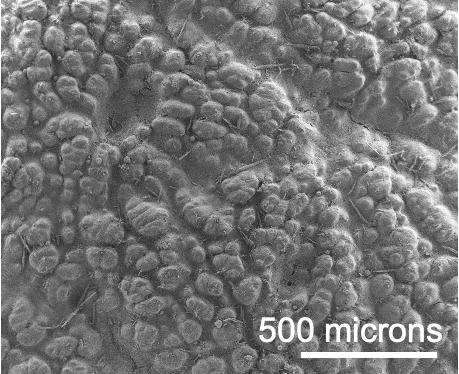

Supplement: obaa049_Supplementary_Data [file obaa049_supplementary_data.zip › Figure S1.tiff]
